# Should we prioritise children 6–23 months of age for vitamin A supplementation? Case study of West and Central Africa

**DOI:** 10.1136/bmjnph-2023-000711

**Published:** 2024-02-06

**Authors:** Arnaud Laillou, Simeon Nanama, Alemayehu Hussen, John Ntambi, Kaleab Baye

**Affiliations:** 1 Nutrition Section, UNICEF West and Central Africa Region, Dakar, Senegal; 2 Center for Food Science and Nutrition, Addis Ababa University, Addis Ababa, Ethiopia; 3 Research Center for Inclusive Development in Africa, Addis Ababa, Ethiopia

**Keywords:** Nutrient deficiencies, Malnutrition

## Abstract

**Background:**

Vitamin A (VA) supplementation has been associated with reductions of all-cause child mortality. Child mortality amenable to VA, particularly related to infectious diseases, may be age dependent; hence, the beneficial effect of VA supplementation may differ between younger and older children. We aimed to estimate the all-cause child mortality disaggregated by younger and older than 2 years of age and estimate the contribution of VA supplementation in preventing child death in West and Central Africa.

**Methods:**

Using the most recent (post-2010) cross-sectional Demographic and Health Surveys and Multiple Indicator Cluster Surveys, we analysed child-level data (n=187 651) from 20 West and Central African countries. Age-specific (all-cause) mortality rates were estimated using survival analyses. Age-specific VA supplementation coverage was linked with the age-specific all-cause child mortality to estimate the contribution of the supplementation in averting child death.

**Results:**

The cost per averted child death was also estimated using an average cost of US$1.2/child and VA supplementation coverage which ranged from 14% in Cote d’Ivoire to 81% in the Gambia. About 75% of the under-5 mortality occurred in the first 2 years of life. The share of excess (all-cause) mortality averted by VA supplementation was significantly higher in the first 2 years of life. A mean reduction of 7.1 deaths/1000 live births was estimated for children 6–23 months, compared to a reduction of 2.5 deaths/1000 live births for older children (24–59 months). The mean cost/averted child death for the 20 countries was 2.8 times lower for the 6–23 than the 24–59 months age group.

**Conclusion:**

Prioritising VA supplementation for children in the first 2 years of life could be more cost-effective than when implemented among 6–59 months of age.

**WHAT IS ALREADY KNOWN ON THIS TOPIC**

- Vitamin A supplementation reduces the overall risk of death in children 6–59 months of age.
- A disaggregated analysis suggests that the effect of vitamin A supplementation may vary by child age and geography.

**WHAT THIS STUDY ADDS**

- The majority of child deaths in West and Central Africa occur in children aged 6–23 months.
- Vitamin A supplementation is more effective and less costly in averting child deaths in children aged 6–23 months compared with older children.

**HOW THIS STUDY MIGHT AFFECT RESEARCH, PRACTICE OR POLICY**

- The findings suggest that additional benefits are likely with a focus of vitamin A supplementation in children 6–23 months than the current targets (6–59 months).
- Implementation research on when, where and how to make this age prioritisation is needed.

## Introduction

Vitamin A (VA) supplementation is and remains one of the key nutrition interventions implemented at scale.^1^ Every year, billions of VA capsules are distributed through the health system to reduce VA deficiency, reducing child morbidity and averting child death.^2^ However, with the increasing number of countries implementing interventions such as oil fortification and biofortification that can prevent VA deficiencies, continued VA supplementation is now primarily justified by its beneficial effect on reducing child morbidity and mortality^3 4^ Indeed, the anti-infectious vitamin was named after its immune-regulating function and beneficial effect in reducing diarrhoea and measles mortality.^5^

The most recent meta-analysis by Imdad *et al* suggests that VA supplementation reduces the overall risk of death by 12% for children aged 6–59 months.^6^ The certainty of the evidence was rated as high for mortality reductions related to diarrhoea, but to a lesser extent for measles and meningitis. A disaggregated analysis also suggested that the effect of VA supplementation may vary by child age and geography (eg, Asia vs Africa).^6^ Furthermore, a recent study by Baye *et al* 4 indicated that the estimated averted child deaths related to VA supplementation are highly variable among countries in the sub-Saharan African region. This can be partly explained by varying levels of all-cause mortality and VA supplementation-amenable child morbidity and mortality. In addition, the age distribution of child morbidity and mortality can significantly influence the effectiveness of the intervention, as VA supplementation is likely to avert more child deaths in age groups where infection-related morbidity and mortality occur the most.^7^

Economic downturns and the low and declining coverage of VA supplementation are raising difficult questions about how best to use the limited funds available to implement the supplementation programmes. Both from an economic and programme effectiveness standpoint, focusing the VA supplementation programme on a narrower age range of 6–23 months may be promising if the bulk of child death and health interventions are concentrated in this age group.^8^ However, the pros and cons of such a prioritisation should be evaluated against its impact on child mortality outcomes.

In West and Central Africa (WCA), over 1.8 million children under 5 years of age died in 2019.^9^ A non-negligible share of these deaths could have been prevented with optimal and effective coverage of VA supplementation, but coverage remained not only suboptimal but also showed a declining trend in several countries.^10^ Revitalising VA supplementation programmes is timely, but the potential impact of such prioritisation needs to be investigated. The current study aimed to disaggregate child mortality estimates for the West and Central African region as well as estimate the potential impact of focusing VA supplementation on averting death in various age groups in WCA in order to improve VA supplementation programming.

## Methodology

### Data source

The study used the most recent Demographic and Health Survey (DHS) and Multiple Indicator Cluster Surveys (MICS) conducted in WCA. Countries that did not have a nationally representative household survey after 2010 as well as those with incomplete data on date of birth, age at death and VA supplementation coverage were excluded. A total of 20 countries (16 from the DHS and 4 from MICS) out of the 23 countries in the WCA region met the above criteria. This yielded a total sample size of 187 651 children with complete data on VA supplementation (online supplemental figure S1 and online supplemental table S1). Data on child mortality and population distribution were extracted from the United Nations Inter-Agency Group for Mortality Estimation (UN IGME) and the UN Population Division, respectively.

10.1136/bmjnph-2023-000711.supp1 Supplementary data

10.1136/bmjnph-2023-000711.supp2 Supplementary data

### Patient and public involvement

The study did not have patient and public involvement.

### Mortality estimates by age

First, the total number of child death was estimated from the UN IGME. Age‐specific mortality rates were then estimated. Age-specific mortality rates are traditionally estimated by DHS and MICS using synthetic cohort life tables, in which mortality probabilities are first computed for smaller age categories based on a real cohort experience. However, this approach is confronted with incomplete cohorts, which affects its accuracy. Instead, we estimated the age-specific mortality using survival analyses as described by Victora *et al*.^11^ The survival analyses compute the death over person-years (m-rates) first, which are then converted to probabilities (q-rates) by calculating the average time spent by those who died in the interval, as described in more details in Victora *et al*.^11^ Age-specific child mortality was estimated for the following age groups: 0–5, 6–11, 12–23 and 23–59 months.

### VA supplementation coverage and reductions in all-cause child mortality, by age

Age-specific VA supplementation coverage was calculated using the most recent surveys. The coverages were then linked to the calculated age-specific all-cause child mortality at country level to estimate the supplementation’s effectiveness in lowering all-cause child mortality. The study used estimates of all-cause mortality reductions of 41% (6–11 months) and 32% (12–59 months) related to the implementation of VA supplementation (vs no implementation), based on estimates from Imdad *et al*’s study.^6^

For each country, the number and proportion of child deaths averted were calculated as follows:

Death averted=coverage×effectiveness×affected proportion …. (1).

Where,

Coverage: VA supplementation coverage from the most recent DHS/MICS survey (scenario 1) or a hypothetical near-universal coverage of ≥90% (scenario 2).

Effectiveness: all-cause child mortality reductions associated with VA supplementation, based on Imdad *et al*’s study.^6^

Affected proportion: proportion of the population in the age range considered.

### Cost of VA supplementation per child averted per year

Several studies that estimated the cost of VA supplementation programmes were examined. Cost estimates (per child/year) were available for Senegal (US$1.25), Cameroon (US$1.14) and India (US$1.20), leading to a mean cost of US$1.2/child/year, which corresponded to the UNICEF estimates for the regions of South Asia and Sub Saharan Africa.^2 12–14^

With a coverage of 90%, the cost per averted child death was estimated for each country:

$$costperavertedchilddeath=\left(\frac{Avertedcases}{population\times cost\times coverage}\right)$$

Where,

Averted cases: number of child deaths averted considering a 90% coverage.

Population: total population of the targeted age groups.

Cost: estimated cost for the delivery of VA, including supply and modalities to administer the vitamin to children.

Coverage: using 90% coverage.

## Results

**Figure 1:**
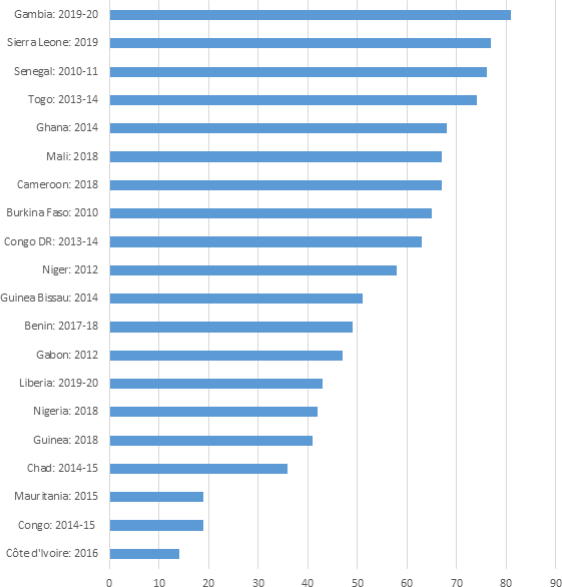
Vitamin A supplementation coverage among children 6–59 years of age in selected West and Central African countries.

Our analyses of the latest DHS/MICS data included 187 651 children with data on coverage of VA supplementation from 20 West and Central African countries. VA supplementation coverage ranged from 14% in Cote d’Ivoire to 81% in the Gambia (figure 1). 8 of the 20 countries had coverage less than 50%, and only 1 country (Gambia) had coverage above 80%. With these coverage levels, the 20 countries saved an estimated 234 505 children’s lives per year (table 1). A scale-up of VA supplementation to ≥90% would have saved an additional 107 981 child lives (342 486 lives).

**Table 1:** Comparison of actual mortality rates versus estimated mortality rates in absence of preventive vitamin A supplementation by age groups

Country	Coverage of VAS	Effectiveness	Estimated reduction with actual coverage	# of children deaths	# of children deaths without current coverage	# of lives saved with the current coverage	# of children deaths with 90% coverage	# of lives saved at 90% coverage
Children aged 6–11 months								
Burkina Faso, 2010	0.65	0.41	0.27	14 146	19 286	5140	12 696	6590
Benin, 2018	0.49	0.41	0.20	5828	7293	1465	4848	2445
Congo DR, 2014	0.63	0.41	0.26	68 011	91 696	23 685	60 482	31 214
Cameroon, 2018	0.67	0.41	0.27	8587	11 839	3252	7777	4062
Gabon, 2012	0.47	0.41	0.19	558	691	133	460	231
Ghana, 2014	0.68	0.41	0.28	4377	6069	1692	3982	2087
Gambia, 2020	0.81	0.41	0.33	405	606	201	390	216
Guinea, 2018	0.41	0.41	0.17	6905	8300	1395	5518	2782
Liberia, 2020	0.43	0.41	0.18	2063	2505	442	1665	840
Mali, 2018	0.67	0.41	0.27	9158	12 626	3468	8294	4332
Nigeria, 2018	0.42	0.41	0.17	126 871	153 263	26 392	101 903	51 360
Niger, 2012	0.58	0.41	0.24	14 602	19 158	4556	12 686	6472
Sierra Leone, 2019	0.77	0.41	0.32	5421	7922	2501	5132	2790
Senegal, 2011	0.76	0.41	0.31	3586	5209	1623	3380	1829
Chad, 2015	0.36	0.41	0.15	13 268	15 565	2297	10 330	5235
Togo, 2014	0.74	0.41	0.30	2449	3516	1067	2288	1228
Congo, 2015	0.19	0.41	0.08	787	853	66	558	295
Cote d'Ivoire, 2016	0.14	0.41	0.06	7481	7937	456	5150	2787
Guinea Bissau, 2014	0.51	0.41	0.21	580	733	153	487	246
Mauritania, 2015	0.19	0.41	0.08	917	994	77	650	344
Children aged 12–59 months								
Burkina Faso, 2010	0.63	0.32	0.20	12 454	15 599	3145	11 378	4221
Benin, 2018	0.53	0.32	0.17	33 184	39 961	6777	29 255	10 706
Congo DR, 2014	0.72	0.32	0.23	22 264	28 929	6665	20 982	7947
Cameroon, 2018	0.53	0.32	0.17	21 959	26 444	4485	19 359	7085
Gabon, 2012	0.55	0.32	0.18	71 764	87 092	15 328	63 726	23 366
Ghana, 2014	0.65	0.32	0.21	944	1192	248	868	324
Gambia, 2020	0.53	0.32	0.17	1260	1517	257	1111	406
Guinea, 2018	0.41	0.32	0.13	13 683	15 749	2066	11 538	4211
Liberia, 2020	0.47	0.32	0.15	15 379	18 101	2722	13 263	4838
Mali, 2018	0.68	0.32	0.22	3160	4039	879	2938	1101
Nigeria, 2018	0.46	0.32	0.15	26 791	31 415	4624	23 019	8396
Niger, 2012	0.60	0.32	0.19	40 490	50 111	9621	36 603	13 508
Sierra Leone, 2019	0.68	0.32	0.22	314 170	401 547	87 377	292 052	109 495
Senegal, 2011	0.90	0.32	0.29	9846	13 829	3983	9846	3983
Chad, 2015	0.46	0.32	0.15	7397	8674	1277	6356	2318
Togo, 2014	0.83	0.32	0.27	6284	8557	2273	6143	2414
Congo, 2015	0.26	0.32	0.08	3181	3470	289	2530	940
Cote d'Ivoire, 2016	0.17	0.32	0.05	29 540	31 239	1699	22 639	8600
Guinea Bissau, 2014	0.68	0.32	0.22	2019	2581	562	1877	704
Mauritania, 2015	0.26	0.32	0.08	1812	1976	164	1441	535

VAS, Vitamin A Supplementation.

**Figure 2:**
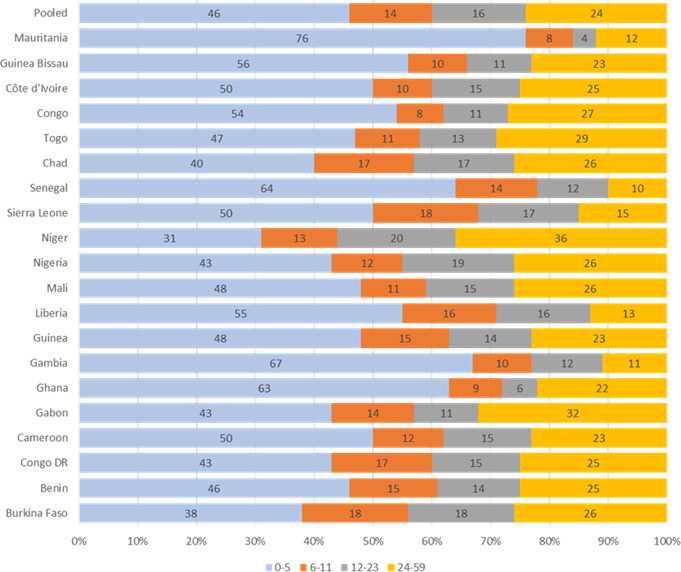
Per cent distribution of child mortality (0–59 months) by age group.

**Figure 3:**
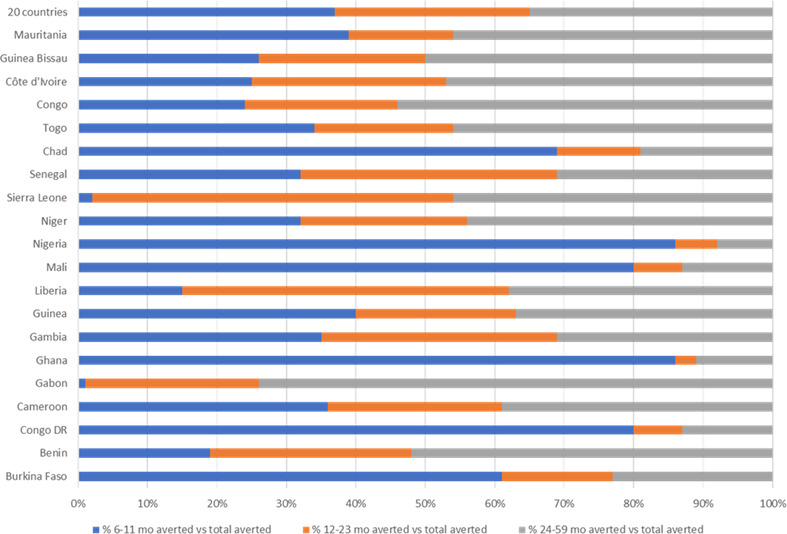
Per cent share of averted deaths among children 6–59 months by age group.

Figure 2 presents the distribution of under-5 child mortality by smaller age groups. The pooled analysis of data from the 20 countries suggests that about 75% of the under-5 mortality occurs in the first 2 years of life (0–23 months of age). Child mortality among infants 0–23 months of age ranged from 64% in Niger to 90% in Senegal. Consequently, the share of excess all-cause mortality averted by VA supplementation was significantly higher between 6 and 23 months compared with later, 24–59 months (figure 3). The country with the highest proportion of VA supplementation-averted child deaths between 6 and 23 months was Nigeria, where above 90% of child mortality avertable by VA supplementation occurred. The lowest VA supplementation-averted child death was reported for Gabon, where a little over 25% of child deaths that could be averted by VA supplementation occurred from 6 to 23 months.

Under 90% coverage, the mortality reduction rate (per 1000 live births) and the cost per child death avoided were calculated (table 2). In line with the absolute number of child deaths averted in the 20 countries, a mean reduction of 7.1 deaths per 1000 live births was estimated for children 6–23 months of age, compared with a reduction of 2.5 deaths per 1000 live births for children 24–59 months of age. The average cost per averted child death for the 20 countries was about three times (×2.8) lower for the 6–23 age group than the 24–59 age group.

**Table 2:** Estimated cost per averted death (in USD) for 90% coverage and child averted by vitamin A supplementation per 1000 children (6–59 months)

For cost 1.2 USD/child for VAS					
Country	Cost/averted deaths among children 6–59 months (in USD)	Cost/averted deaths among children 6–23 months (in USD)	Cost/averted deaths among children 24–59 months (in USD)	Child deaths averted 6–23 months per 1000 children 59 months per 1000 children	Child deaths averted 24 months
Benin	148.2	65.7	424.3	16.4	2.5
Burkina Faso	328.2	233.2	415.8	4.6	2.6
Cameroon	372.6	145.4	1893.0	7.4	0.6
Chad	398.8	224.1	672.1	4.8	1.6
Republic of Congo	666.6	868.3	595.8	1.2	1.8
DR Congo	107.9	41.3	646.4	26.1	1.7
Cote d'Ivoire	1425.6	706.6	3025.9	1.5	0.4
Gabon	13.4	7.1	24.1	152.7	44.8
Gambia	671.9	369.8	1164.9	2.9	0.9
Ghana	1716.0	664.7	8752.0	1.6	0.1
Guinea	307.3	114.0	2530.6	9.5	0.4
Guinea Bissau	320.9	193.7	482.7	5.6	2.2
Liberia	132.3	83.4	189.7	13.0	5.7
Mali	682.6	339.0	1447.3	3.2	0.7
Mauritania	793.3	332.5	2757.8	3.2	0.4
Niger	252.3	161.3	359.2	6.7	3.0
Nigeria	576.3	427.2	703.3	2.5	1.5
Senegal	450.0	287.1	633.8	3.8	1.7
Sierra Leone	10.3	7.0	13.6	155.0	79.2
Togo	338.6	213.1	485.9	5.1	2.2
Total 20 countries	265.4	152.0	432.4	7.1	2.5

## Discussion

Analysing nationally representative data from 20 West and Central African countries, this study showed that the coverage of VA supplementation was highly variable between countries but remained low relative to the WHO minimum target of 80%. Under-5 child mortality was concentrated in the first 2 years of life, resulting in the greatest VA supplementation-related mortality reductions for younger (6–23 months) children versus older (24–59 months). The cost of life savings per child was also much lower for younger children (6–23 months).

The reduction in under-5 child mortality witnessed over the past decades, the increasing availability of alternative VA interventions (eg, fortification), the perceived risk of excessive VA intake and the increasing competition over limited resources for other programmes have led to a call for a fresh look at VA supplementation programmes.^15^ The study by Baye *et al* 4 recently showed that the excess child mortality that can be averted by VA supplementation has declined over the years, but also illustrated that it remains significant for several countries in sub-Saharan Africa. According to the study, some countries could benefit from a scaleback or a more targeted approach. Indeed, another finding suggested that perhaps focusing resources on younger children could help increase effective coverage through more focus and better integration of VA supplementation with other preventive nutrition and health interventions.^7^

Our study has shown that about two-thirds of child deaths in the West and Central African countries included in the analyses occur for children 6–23 months, a finding in line with a recent global analysis.^8^ The recently updated meta-analyses also showed that all-cause mortality reductions related to VA supplementation are slightly higher for younger (6–11 months) than older (12–59 months) children (32%).^6^ The cost per child death avoided was also in favour of prioritising the programme for younger children (6–23 months) over older children (three times lower). Further reductions in delivery costs can be expected with shifts to routine programme delivery through the health system, though it has been difficult to estimate the savings.^13^ In addition, campaign fatigue is already leading countries and donors to transition to routine delivery. Although theoretically more sustainable, such shifts have already unmasked the weaknesses and inequities of the health system, where the more remote and vulnerable often have limited access.^15 16^ In addition, in places where VA supplementation was bundled with polio vaccination, coverage also showed a drop due to the phasing out of the polio vaccination campaigns as polio irradiation goals were getting within reach. Altogether, if shifts to routine delivery are the way forward, careful planning and alignment with other nutrition and health interventions will be critical. However, our results suggest the possibility to increase VA supplementation coverage and cost-effectiveness in reducing child mortality if the intervention is focused to children 6–23 months of age.

This study has several limitations that need to be considered when interpreting our findings. First, this is a cross-sectional study; hence, causality cannot be established. Our estimates of excess mortality avoidable through VA supplementation, on the other hand, were based on evidence from meta-analyses of randomised controlled trials. Second, the study used VA supplementation coverage estimates from population-based surveys such as DHS/MICS, which only capture exposure over the previous 6 months. Given that the relative risk of child mortality related to VA supplementation is established for two doses, the finding assumed a similar coverage for the second semester. Third, the cost of delivery of VA supplementation was primarily estimated for programmes delivered through campaigns; hence, estimates for VA supplementation delivered through the routine health system are likely to be overestimated.

Notwithstanding the above limitations, this study showed that a higher proportion of child mortality in the region that could be prevented by VA is concentrated in the first 2 years of life, between 6 and 23 months. Prioritising VA supplementation for children in the first 2 years of life could be more cost-effective than when implemented among 6–59 months of age. While considering the lowering of the age range of the intervention, it is also important to strengthen links with the health system, and address existing inequalities in access to healthcare. With changing diets, disease epidemiology, overlapping interventions and shifts in VA supplementation delivery and targets, ensuring close monitoring and evaluation will be critical.

## Data Availability

Data are available on reasonable request. Data can be accessed from the DHS programme on request.
