# Supplementary figures and images for: Should we prioritise children 6–23 months of age for vitamin A supplementation? Case study of West and Central Africa
Source: BMJ Nutr Prev Health. 2024 Feb 6;7(1):88–94. doi: 10.1136/bmjnph-2023-000711 (PMC11221273; doi:10.1136/bmjnph-2023-000711)

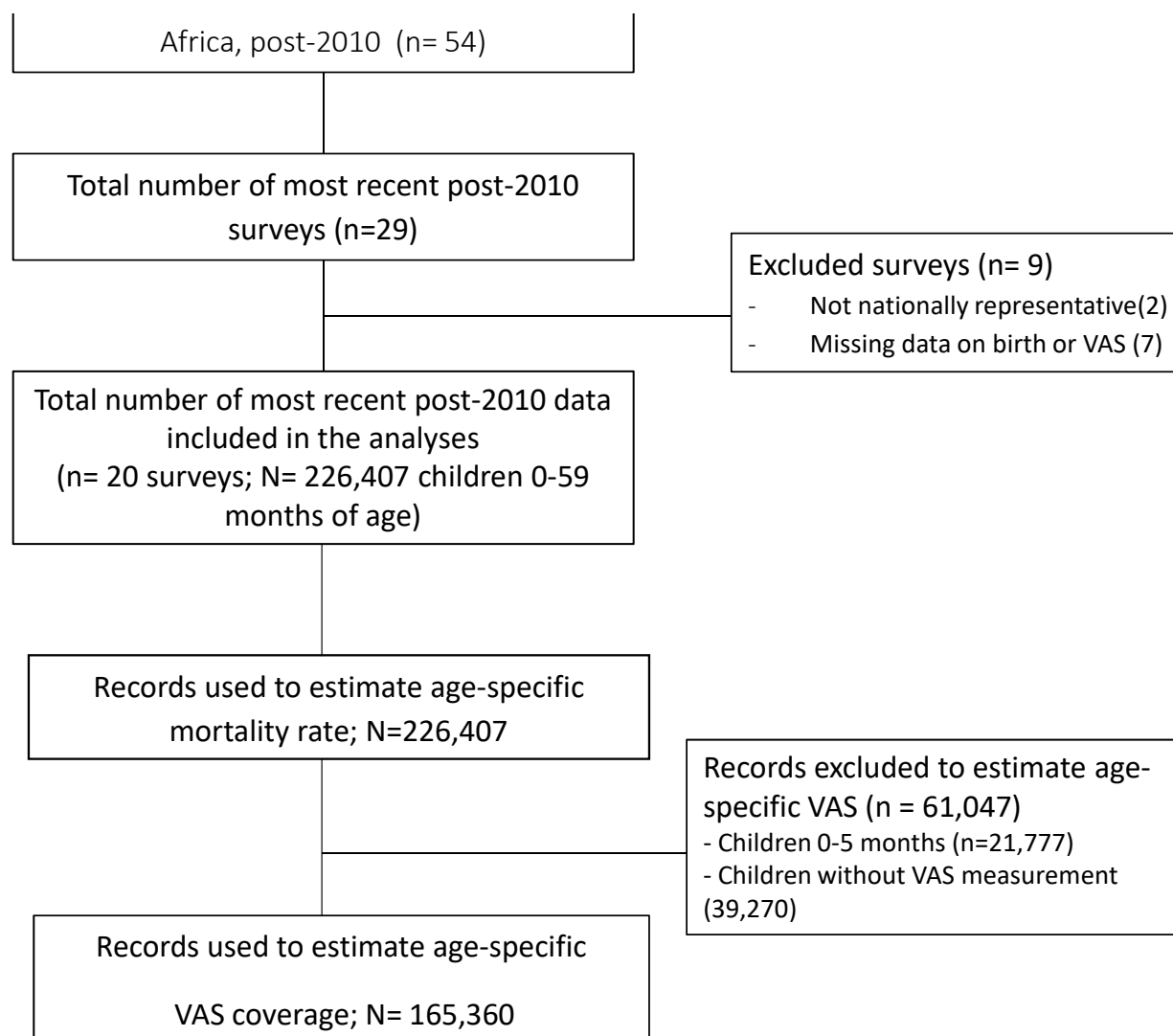

**Figure S1** Flowchart showing participants included in the analyses

Supplement: Supplementary data [file bmjnph-2023-000711supp001.pdf]
