# Supplementary material for: Should we prioritise children 6–23 months of age for vitamin A supplementation? Case study of West and Central Africa
Source: BMJ Nutr Prev Health. 2024 Feb 6;7(1):88–94. doi: 10.1136/bmjnph-2023-000711 (PMC11221273; doi:10.1136/bmjnph-2023-000711)
Supplement: Supplementary data [file bmjnph-2023-000711supp002.pdf]

Table S1 – Survey included and number of Children by country

Country: year of survey	Total Number of children	Number of children measured for VAS	Coverage of Vitamin A supplementation (%)
**Burkina Faso: 2010**	15,044	13,716	65
**Benin: 2017-18**	13,589	12,651	49
**Congo DR: 2013-14**	18,716	17,167	63
**Cameroon: 2018**	9,733	9,085	67
**Gabon: 2012**	6,067	5,747	47
**Ghana: 2014**	5,884	5,595	68
**Gambia: 2019-20**	8,362	7,927	81
**Guinea: 2018**	7,951	7,273	41
**Liberia: 2019-20**	5,704	5,245	43
**Mali: 2018**	9,940	9,275	67
**Nigeria: 2018**	33,924	30,713	42
**Niger: 2012**	12,558	11,602	58
**Sierra Leone: 2019**	9,899	9,063	77
**Senegal: 2010-11**	6,935	6,602	76
**Chad: 2014-15**	18,623	16,729	36
**Togo: 2013-14**	6,979	6,525	74
**Congo: 2014-15**	9,169	2,889	19
**Côte d'Ivoire: 2016**	9,094	4,291	14
**Guinea Bissau: 2014**	7,573	3,596	51
**Mauritania: 2015**	10,663	1,960	19
**Total**	**226,407**	**187,651**	
